# Amyloid-related imaging abnormalities after immunotherapy: How might brain microvascular basement membrane components be involved?

**DOI:** 10.1177/13872877261467705

**Published:** 2026-07-11

**Authors:** Harry V. Vinters, Shino Magaki

**Affiliations:** 1 Department of Pathology & Laboratory Medicine (Neuropathology), 12222 David Geffen School of Medicine at UCLA, & Ronald Reagan UCLA Medical Center, Los Angeles, CA, USA; 2 Department of Neurology, 12222 David Geffen School of Medicine at UCLA, & Ronald Reagan UCLA Medical Center, Los Angeles, CA, USA

**Keywords:** Alzheimer's disease, neurodegeneration, neurodegenerative diseases, neuropathology

## Abstract

Basement membrane components are integral to the physiologic function of cerebral microvessels. Immunotherapy (including by the use of lecanemab) in Alzheimer's disease patients may result in vascular complications identified by neuroimaging (ARIA-E and ARIA-H). This pilot study (on a relatively small number of human brain specimens) suggests one mechanism might be through the effects of lecanemab on a collagen component of said microvessel walls. It utilizes microvessels isolated from human brains (of Alzheimer's disease patients) and maintained in a viable state, to examine this mechanism using novel biochemical and molecular approaches. These yield preliminary evidence of how lecanemab may influence a specific component of the cerebral microvasculature and suggest other studies that may be used to address this important question.

Basement membrane (BM) components are an integral part of cerebral microvessel walls, vital to normal physiologic functions of these structures, composed of the neurovascular unit including the blood-brain barrier.^1^ They develop pronounced, sometimes age-related changes over time, and contribute to the two most common cerebral ‘angiomyopathies’, arteriolosclerosis and cerebral amyloid angiopathy (CAA).^2,3^ The basis for the present pilot study^4^ is the known close association of vascular BM components (especially collagens, e.g., collagen IV) with amyloid-β (Aβ) deposits within brain microvessels.^1,5^ The authors hypothesize that biochemical and molecular abnormalities of this collagen—abundantly expressed within vascular BM—may be an important component of vascular complications of Aβ immunotherapy, i.e., amyloid-related imaging abnormalities (ARIA) including severe cerebral edema (ARIA-E) and intraparenchymal hemorrhage (ARIA-H). Unfortunately, relatively few neuropathologic studies (compared to the abundance of radiographic investigations) have addressed ARIA pathogenesis; the two excellent reviews on ARIA^6,7^ highlight the importance of CAA in ARIA pathogenesis (including excellent and meticulous clinicopathologic investigations from James Nicoll's laboratory in the United Kingdom). ARIA is not a rare or trivial complication of immunotherapy: either ARIA-E or ARIA-H occur in approximately 13–16% of treated individuals. The majority of individuals with ARIA are asymptomatic with symptomatic cases, most commonly headache, confusion, nausea or vomiting, and visual or gait disturbances, ranging from 6 to 39% depending on particular therapeutic and dose; however, rare cases of severe neurologic symptoms and deaths thought to be related to cerebral hemorrhage have been reported.^8,9^

The biochemical and molecular experiments described are straightforward and suggestive of microvessel dysfunction in ARIA. Although no definite causal evidence is presented, the results warrant future mechanistic studies. Of special interest is the fact that they were performed on microvessels isolated from autopsy brain specimens with ‘high Alzheimer disease neuropathologic change’ (ADNC, including frequent CAA) and retained in a relatively viable state for several days after isolation. None of the patients, brains from which were studied, had been exposed to lecanemab. A subset of microvessels from some donors showed collagen IV changes that might result in microvascular injury (microangiopathy) when exposed to lecanemab. The fact that only a subset showed these changes likely reflects variability in local Aβ deposition, vascular vulnerability, and microenvironmental factors. The study used a relatively small, though well-characterized, number of specimens, mainly from patients in their 80 s. Because BM components in cerebral microvessel walls are in a dynamic state and probably undergo age-related changes, the findings may not be predictive of what might be seen in younger immunotherapy-treated AD subjects or those with familial AD. Furthermore, the pathogenesis of ARIA is likely multifactorial, involving other pathways such as vascular Aβ clearance, inflammation, endothelial dysfunction, and *APOE*-related vascular vulnerability.^8^ As well, the experiments are ‘acute/subacute’ in terms of their time frame. ARIA tends to occur in individuals as a late complication of treatment, during which microvascular elements have been exposed to immunotherapeutic agents repeatedly.

One encouraging outcome of the experiments is that they suggest the possibility of using autopsy derived cerebral microvessels (and possibly other elements) as a substrate for sophisticated biochemical and molecular investigations. While mouse and rat experiments can provide clues to disease (or therapy) related mechanisms in neurodegenerative disease, only the ‘human specimen’ provides a global picture of Alzheimer's disease or any other neurodegenerative disease. For one of us (HVV) who early in his career isolated human brain microvascular endothelium and smooth muscle cells from surgical specimens,^7^ it is gratifying to see similar techniques being effectively applied to autopsy brain tissue. The pathogenesis of ARIA-E and ARIA-H is poorly understood. The reported studies provide a ‘roadmap’ to molecular and biochemical investigations that may provide a more ‘granular’ insight into how and why these complications occur.
